# Primary Clear Cell Adenocarcinoma of the Cervix: A Clinical Analysis of 18 Cases without Exposure to Diethylstilbestrol

**DOI:** 10.1155/2019/9465375

**Published:** 2019-03-26

**Authors:** Dongying Wang, Chunhua Zhao, Li Fu, Yang Liu, Weiyang Zhang, Tianmin Xu

**Affiliations:** Department of Obstetrics and Gynecology, Second Hospital of Jilin University, Changchun, Jilin 130000, China

## Abstract

**Objectives:**

Cervical clear cell adenocarcinoma (CCAC) is a rare malignant tumor with independent biological behavior in the female reproductive system. In this report, we collect the clinical and histopathological characteristics of 18 CCAC patients without exposure to diethylstilbestrol (DES) and conduct relevant clinical analysis.

**Methods:**

We retrospectively analyzed the clinical data of 18 patients with CCAC who were diagnosed and treated from January 2009 to August 2017 in the Second Hospital of Jilin University.

**Results:**

A total of 18 patients were enrolled. The age of patients ranged from 37 to 74 years with the peak incidence between 45 and 55 years. The median age was 53 years. The most common symptom was vaginal bleeding (66.7%, 12/18). The most common type of lesion was the endocervical type (66.7%, 12/18). The negative rate of human papillomavirus (HPV) examination was 88.9% (8/9). Based on the staging criteria of the International Federation of Gynecology and Obstetrics (FIGO) cervical cancer clinical stage in 2018, 55.6% patients were stage I (*n*=10), 16.7% were stage II (*n*=3), 22.2% were stage III (*n*=4), and 5.6% were stage IV (*n*=1). Seventeen patients underwent surgery; 64.7% (11/17) of cases showed infiltration of the entire layer of the cervix, pelvic lymph node (PLN) metastasis was observed in 4 patients (26.7%, 4/15), endometrium metastasis was observed in 4 patients (25%, 4/16), and 13 patients (72.2%, 13/18) were diagnosed at an early stage (stage IB1-IIA2). Fifteen patients' immunohistochemistry indicated that napsin A, CK7, CK (AE1/AE3), and PAX-8 were positive, and p53, p16, ER, and vimentin were expressed to different degrees. Follow-up data were obtained in 13 patients (72.2%, 13/18). One patient died of recurrence 5 months after surgery, and the other patients' progression-free survival (PFS) ranged from 9 to 59 months. Tumor size (>4 cm), tumor stage (FIGO IIA2-IV), PLN, and endometrium metastasis had negative effects on PFS (*P* < 0.05).

**Conclusions:**

CCAC is a highly invasive malignant tumor, whose pathogenesis may not be associated with HPV infection. Radical hysterectomy combined with chemotherapy (paclitaxel + platinum) has the ideal short-term curative effect. In the future, larger samples of clinical data are required to confirm these insights.

## 1. Introduction

CCAC is a rare pathological type of cervical cancer that is likely to differentiate toward endometrial adenocarcinoma [1]. In 1971, Herbst et al. [2] first reported that CCAC occurs in women whose mothers were exposed to DES during pregnancy. However, Kaminski and Maier [3] revealed that CCAC can also occur without exposure to DES in 1983. In the post-DES era, the incidence of CCAC has decreased, accounting for approximately 4% to 9% of all cervical adenocarcinomas (AC) [1].

Currently, only a few cases of CCAC have been reported, and there is limited information on clinical behavior, histopathology features, patient management, and prognosis about this tumor and lack of multisample case reports for Asian women. In this study, we analyzed 18 CCAC patients without history of DES exposure, summarized their clinicopathological features, and performed survival analysis to provide relevant reference information for clinical studies of CCAC.

## 2. Materials and Methods

We reviewed the clinical data of 18 cases of CCAC patients, who were diagnosed and treated at the Second Hospital of Jilin University from January 2009 to August 2017. All of the cases were confirmed to be CCAC by two or more pathologists through pathological examination. The research was approved by the Institutional Review Board of the Second Hospital of Jilin University.

We used the FIGO cervical cancer clinical staging system. The clinical fundamental characteristics included age, marital and menstrual history, clinical symptoms, HPV-DNA and TCT tests, and histological and immunohistochemical data. Operative techniques included radical hysterectomy, bilateral salpingo-oophorectomy, and pelvic lymphadenectomy with or without paraaortic lymphadenectomy. Further treatment by radiation or chemotherapy was undertaken when prognostic factors were unfavorable (tumor size >4 cm; cervical invasion >2/3; lymphovascular space involvement (LVSI); PLN or endometrial/uterine corpus metastasis), discretion of the doctor in charge, and the actual institutional practices at the time. For follow-up data, PFS was calculated. In the first 2 years, the follow-up period was 3 months. In the next 3 to 5 years, the follow-up period was 6 months, and in the subsequent years, the follow-up period was 12 months. IBM SPSS 25.0 was used for statistical analysis. Kaplan–Meier curves were used to describe survival, and the log-rank test was performed to compare the survival of different groups. *P* values less than 0.05 were considered statistically significant (*P* < 0.05).

## 3. Results

**Figure 1:**
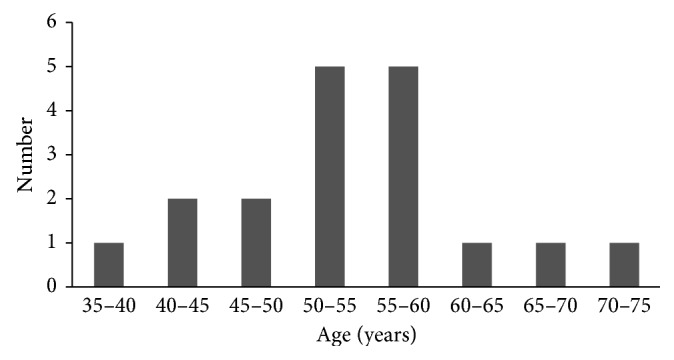
Age distribution of patients with CCAC (*n*=18).

We summarized 18 patients with CCAC. The detailed clinical information is shown in Table 1. The median age was 53 years (range from 37 to 74 years) with a peak incidence between 45 and 60 years (Figure 1). Eleven patients (61.1%) were postmenopausal at the time of diagnosis. Two patients (11.1%) were childless. All of the patients denied a history of exposure to DES. The most common clinical symptom was irregular vaginal bleeding (66.7%, 12/18), and other symptoms were contact vaginal bleeding (11.1%, 2/18) and abnormal vaginal discharge (16.7%, 3/18). The endocervical type was the most common type (66.7%, 12/18). There was no significant difference in clinical symptoms between early- and later-stage patients. The tumor size ranged from 1 to 8 cm with a medium size of 3.4 cm. According to FIGO cervical cancer clinical staging in 2018, 55.6% patients were stage I (*n*=10), 16.7% were stage II (*n*=3), 22.2% were stage III (*n*=4), and 5.6% were stage IV (*n*=1). Nine patients underwent HC2 HPV-DNA testing, and only one patient was positive (11.1%). Six patients underwent cervical ThinPrep Cytologic testing (TCT), 4 patients were positive (66.7%), 2 patients were diagnosed as “adenocarcinomas (AC),” 1 patient was diagnosed as “atypical glandular cells (AGC),” and 1 patient was diagnosed as “malignant cells.” Seven patients underwent serum CA125 testing, and 3 patients were positive (CA125 ≥ 30 *μ*/ml), ranging from 41.7–94 *μ*/ml.

In preoperative histopathological examination (*n*=16), 6 patients were diagnosed as “adenocarcinomas with uncertain pathological type,” 6 patients were diagnosed as “clear cell adenocarcinoma,” and 4 patients were misdiagnosed as “endometrial adenocarcinoma” or “adenosquamous carcinoma.” All preoperative biopsy cases were confirmed by histopathology as CCAC after surgery. Fifteen patients (83.3%) underwent radical hysterectomy, bilateral salpingo-oophorectomy, and pelvic lymphadenectomy with/without paraaortic lymphadenectomy. Two patients underwent neoadjuvant chemotherapy (paclitaxel + carboplatin) for 3 courses. One patient did not undergo surgery after preoperative hysteroscopy and histopathological diagnosis. One patient underwent extrafascial hysterectomy and bilateral salpingo-oophorectomy, because she had undergone colon cancer surgery 3 weeks prior in another hospital. In total, 64.7% (11/17) of cases had infiltration of the entire layer of the cervix. PLN metastasis was found in 4 cases (26.7%, 4/15), endometrium metastasis in 4 patients (25%, 4/16), and thirteen patients (72.2%, 13/18) were diagnosed at an early stage (stage IB1-IIA2). In postoperative immunohistochemical examination (*n*=15), napsin A, CK7, CK (AE1/AE3), and PAX-8 were positive, and p53, p16, ER, and vimentin were expressed to different degrees, while PR, WT-1, P40, CDX2, AFP, CK20, GATA3, CgA, and Syn were negative.

**Figure 2:**
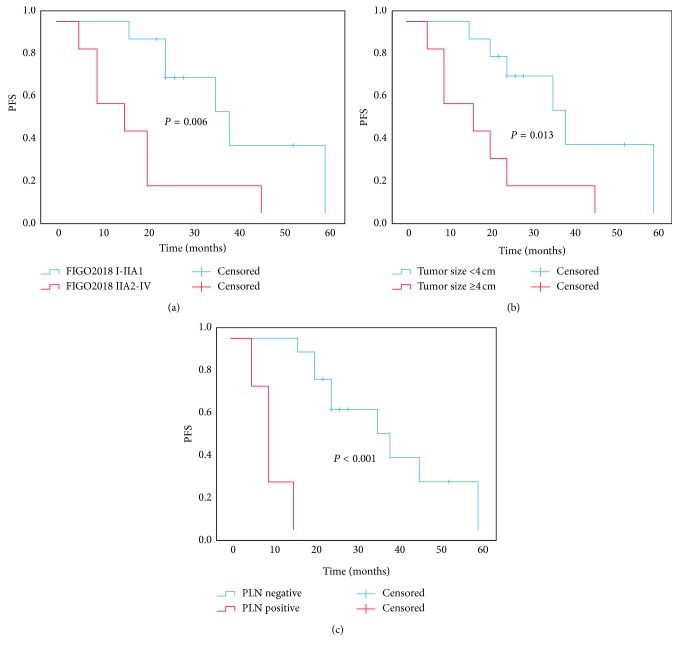
Kaplan–Meier curve of PFS. (a) The PFS for stages I to IIA1 and stages IIA2 to IV (*P*=0.006). (b) The PFS for tumor size <4 cm and ≥4 cm (*P*=0.013). (c) The PFS for PLN metastasis positive and negative patients (*P* < 0.001).

Five patients were lost to follow-up, and their data were censored at the last contact. One patient died of recurrence 5 months after surgery, and the other patients' progression-free survival (PFS) ranged from 9 to 59 months. Kaplan–Meier survival estimates showed that patients with larger tumor size (>4 cm), higher FIGO stage (stage IIA2-IV), PLN metastasis, and/or endometrium metastasis had negative effects on PFS (*P* < 0.05, Figure 2). There was no significant correlation between deeper cervical invasion (more than two-thirds) and prognosis (*P* > 0.05). Other factors, such as age and personal and family history, did not affect prognosis (*P* > 0.05).

Seven of the 11 patients who had risk factors received postoperative adjuvant therapy. One patient received PR (postoperative radiotherapy, median RP 49.5 Gy), 2 patients received NC + PR (neoadjuvant chemotherapy and postoperative radiotherapy, median RP 49.5 Gy), and 4 patients received PPBC + PR (postoperative platinum-based chemotherapy and radiotherapy, median PR 49.5 Gy). Four patients without risk factors also received postoperative adjuvant therapy, and all of them received postoperative chemotherapy. Chemotherapy regimens were cisplatin or carboplatin with paclitaxel from day 1 to day 3 in each cycle, and 5 or 6 cycles were administered at 3-week intervals. There were 4 patients with recurrence, two (50%) in vaginal stumps, and two (50%) in pelvic and paraaortic lymph nodes. One patient who had three risk factors died of recurrence 5 months after surgery.

## 4. Discussion

CCAC in females is a rare cervical malignancy accounting for 3%–10% of all cervical adenocarcinomas [4, 5], and there are few reports on the clinicopathological features, diagnosis, treatment, and prognosis. In this retrospective study, we describe the clinical and histopathological characteristics of 18 patients without exposure to DES.

A comparative analysis showed that the mean age of patients without exposure to DES was 47 years (ranged from 31 to 64 years), and only 2 patients were less than 35 years [1]. Thomas et al. [6] collected 34 cases of CCAC at three centers from 1982 to 2004 and showed that the median age of patients was 53 years, and only 3 patients were younger than 30 years. In our study, the median age was 53 years (range from 37 to 74 years) with a peak incidence between 45 and 55 years, and the result is consistent with Thomas et al. [6] and Reich et al. [1]. Therefore, we believe that primary clear cell adenocarcinoma is no longer a disease that only affects young women. In the post-DES era, CCAC mainly occurs in postmenopausal women.

Pirog et al. [7] reported the incidence of HPV infection in 760 cervical adenocarcinoma cases, and the clear cell type had a lower HPV prevalence at 20%. One case reported synchronous invasive squamous cell carcinoma (SCC) and CCAC in one patient, and HPV 18 was detected in the SCC. However, no HPV was detected in the CCAC [8]. In our study, the incidence of HPV infection was 11.1% (1/9), indicating that the pathogenesis of CCAC may not be related to HPV infection. The most common clinical symptom of SCC is contact vaginal bleeding while CCAC is irregular vaginal bleeding [6], which is consistent with our report (66.7%, *n*=18). Reich et al. [1] reported that 80% of CCAC (15 cases of I B-II B stage) is endocervical type and tends to invade deep into the cervix, and 33% (5/15) invade the uterus. In total, 76% of patients were staged earlier than stage IIA [6]. These data are consistent with our study, as endocervical type was found in 66.7% (12/18), full-thickness cervical invasion was detected in 64.7% (11/17), endometrial/uterine corpus metastasis was observed in 25% (4/16), and 72.2% (13/18) of patients were earlier than stage IIA1. These characteristics may cause a lower positive rate of cervical cytology examination. Thomas et al. [6] reported that only 18% of CCAC patients (6/34) had abnormal Pap tests. However, 66.7% of patients (4/6) had abnormal TCT tests in our study. This discrepancy may be because most of our patients had obvious lesions during gynecological examinations. There are no effective tumor markers for CCAC. Bender et al. [9] indicated that serum CA 125 (≥30 U/mL) is an independent prognostic marker for patients with cervical adenocarcinoma (33% of the 73 patients), which was significantly associated with advanced FIGO stage > IIA (*P*=0.01), larger tumor size >4 cm (*P* < 0.01), and positive pelvic or paraaortic lymph nodes (*P*=0.002). In our study, 3 patients were positive (CA 125 ≥ 30 *μ*/ml). Among them, one patient was lost to follow-up, and the other two patients were negative after postoperative adjuvant therapy. Therefore, serum CA 125 levels may be associated with CCAC, but more cases are necessary to confirm this finding.

CCAC has a higher risk of pelvic or paraaortic lymph nodes, corpus uteri, and parametrial metastasis compared with SCC [1, 6]. Overall ovarian metastasis rates in patients with cervical cancer were between 0.9% and 2.2% [10, 11]. However, the incidence of ovarian metastasis varied in different histologic types, ranging from 0.4% to 1.9% in SCC and from 2.4% to 9.2% in AC [11]. Of note, the independent risk factors for ovarian metastasis of CCAC mainly include [11–14]: (1) histology (AC), (2) age (>45 years), (3) FIGO stage (IB2-IIA, >4 cm), (4) deep stromal invasion (greater than two-thirds), and (5) uterine metastasis. One patient had metastases in the right ovary of 16 patients who underwent bilateral salpingo-oophorectomy. Interestingly, in this patient, histology (AC), age (47 years), tumor size (6 cm), FIGO stage (IIA2), full-thickness cervical invasion, and endometrial metastasis were noted. The Vang et al. [15] analysis of clear cell carcinoma in the female reproductive system (*n*=17) found that the immunohistochemistry was positive for CK7, CAM5.2, 34 beta E12, CEA, Leu-M1, vimentin, bcl-2, p53, and CA 125.ER, and HER-2/neu were expressed to different degrees but negativity for CK20 and PR. In our postoperative immunohistochemical examination (*n*=15), napsin A, CK7, CK (AE1/AE3), and PAX-8 were positive, and p53, p16, ER, and vimentin were expressed to different degrees while PR, WT-1, P40, CDX2, AFP, CK20, GATA3, CgA, and Syn were negative. Clear cell carcinomas appear to have the same immunophenotype in the female reproductive system.

There were limited clinical data on CCAC, and treatment is mainly based on AC and SCC. Surgery is still the main treatment for early CCAC patients (FIGO stage I-II), and patients often choose radical abdominal hysterectomy and pelvic lymphadenectomy with or without paraaortic lymphadenectomy. Baalbergen et al. [16] found that early AC patients (FIGO stage I-II) who underwent radical surgery fared better than those who received radiotherapy. It is recommended that patients with early adenocarcinoma be treated with radical surgery and patients with lymph node positive by MRI or PET-CT be treated with radiotherapy and chemotherapy. Shimada et al. [17] reported that among patients receiving adjuvant radiotherapy, the recurrence rate of CCAC patients (24.6%) was higher than that of SCC (10.5%) (*P*=0.0022). Although AC is less sensitive to radiotherapy than SCC, several studies have confirmed the response of early AC patients with high risk factors to postoperative adjuvant radiotherapy. Stehman et al. [18] found that AC and adenosquamous carcinoma patients (stage IB) with high risk factors are more likely to benefit from postoperative adjuvant radiotherapy than SCC patients, and concurrent weekly cisplatin with radiotherapy significantly improved long-term PFS and OS.

A large study on cervical cancer prognosis (*n*=24,562) found that AC patients at an early stage (IB1-IIA) or advanced stage (IIB-IVA) were more likely to die from their tumors than those with SCC (HR 1.39 and 1.21) [5]. Reich et al. [1] reported that the 5-year survival rate for patients with early stage CCAC was 67%, which was slightly worse than 77% for nonclear cell carcinoma and 80% for SCC, but the difference was not significant. Most patients with CCAC are diagnosed at an early stage (FIGO I-II), and the 3-year and 5-year survival PFS of patients with FIGO I to IIA CCAC were significantly better than patients with stage IIB to IVB CCAC [6, 19]. Both our study and the Yang et al. [20] study confirmed that the risk factors affecting the prognosis of CCAC were larger tumor size (>4 cm), higher tumor stage (stage IIA2-IV), PLN metastasis, and endometrium metastasis. We recommend that patients with the above risk factors undergo adjuvant treatment (platinum-based chemotherapy and radiotherapy) after surgery, even if PR alone or PPBC + PR do not affect the sum survival time for patients with risk factors in our study (*P* > 0.05). Therefore, larger samples and longer clinical follow-up times are required to confirm these insights.

In conclusion, CCAC affects more elderly women in the post-DES era, and its pathogenesis may be unrelated to high HPV infection. For CCAC patients with high risk factors, surgery is the main treatment, and adjuvant radiotherapy and chemoradiotherapy may be effective. Our study was a retrospective study with moderate sample size and limited statistical capacity. Future prospective studies should lead to more information to provide a reference for the clinical diagnosis and treatment of CCAC.

## Figures and Tables

**Table 1:** Patients' clinical characteristics (*n*=18).

Characteristics	*n* (%)
Age distribution	
Median age	53
Age < 45	3 (16.7)
Age ranges from 45 to 60	12 (66.7)
Age ≥ 60	3 (16.7）
Clinical symptoms	
Irregular vaginal bleeding	12 (66.7)
Contact vaginal bleeding	2 (11.1)
Abnormal vaginal discharge	3 (16.7)
Unknown	1
Childbearing history	
Nulliparous	2 (11.1)
Parous	16 (83.3)
Menopausal status	
Premenopausal	6 (33.3)
Postmenopausal	11 (61.1)
Cervical stump	1 (5.6)
Macropathology	
Endocervical type	12 (66.7)
Exogenic type	2 (11.1)
Endogen type	2 (11.1)
Ulcer type	1 (5.6)
Unknown	1 (5.6)
Tumor size (cm)	
≤4 cm	5 (27.8)
>4 cm	11 (61.1)
Unknown	2 (11.1)
HPV-DNA (*n*=9)	
Positive	1 (11.1)
Negative	8 (88.9)
TCT (*n*=6)	
Positive	4 (66.7)
Negative	2 (33.3)
FIGO stage	
I	10 (55.6)
II	3 (16.7)
III	4 (22.2)
IV	1 (5.6)
FIGO stage	
IB1-IIA1	11 (61.1)
IIA2-IV	7 (38.9)
Neoadjuvant chemotherapy	
Yes	2 (11.1)
No	16 (88.9)
Surgery	
Yes	17 (94.4)
No	1 (5.6)
Extent of surgery (*n*=17)	
RAH + BSO + PL	13 (76.5)
RAH + BSO + PL + PAL	2 (11.8)
EH + BSO	1 (5.8)
RECS	1 (5.8)
Cervical invasion (*n*=17)	
<1/3	6 (35.3)
Full-thickness	11 (64.7)
LVSI	
Yes	1 (5.8)
No	16 (94.1)
PLN metastasis (*n*=15)	
Yes	4 (26.7)
No	11 (73.3)
Endometrial/uterine corpus metastasis (*n*=16)	
Yes	4 (25)
No	12 (75)
Ovarian metastasis (*n*=15)	
Yes	1 (6.7)
No	14 (93.3)
Adjuvant treatment (*n*=11)	
PPBC	4 (36.4)
PR	1 (9.1)
PPBC + PR	4 (36.4)
NC + PR	2 (18.2)

BSO, bilateral salpingo-oophorectomy; RAH, radical abdominal hysterectomy; PL, pelvic lymphadenectomy; PAL, paraaortic lymphadenectomy; EH, extrafascial hysterectomy; RECS: radical excision of cervical stump; PPBC: postoperative platinum-based chemotherapy.

## Data Availability

The clinical data used to support the findings of this study are restricted by the ethics board of the Second Hospital of Jilin University in order to protect patient privacy. Data are available from the corresponding author for researchers who meet the criteria for access to confidential data.
